# Interferon lambda is required for interferon gamma-expressing NK cell responses but does not afford antiviral protection during acute and persistent murine cytomegalovirus infection

**DOI:** 10.1371/journal.pone.0197596

**Published:** 2018-05-16

**Authors:** Silvia Gimeno Brias, Morgan Marsden, Jessica Forbester, Mathew Clement, Cordelia Brandt, Katherine Harcourt, Leanne Kane, Lucy Chapman, Simon Clare, Ian R. Humphreys

**Affiliations:** 1 Institute of Infection Immunity, School of Medicine/Systems Immunity University Research Institute, Cardiff University, Cardiff, United Kingdom; 2 Wellcome Trust Sanger Institute, Hinxton, Cambridgeshire, United Kingdom; University of St Andrews, UNITED KINGDOM

## Abstract

Interferon lambda (IFNλ) is a group of cytokines that belong to the IL-10 family. They exhibit antiviral activities against certain viruses during infection of the liver and mucosal tissues. Here we report that IFNλ restricts *in vitro* replication of the β-herpesvirus murine cytomegalovirus (mCMV). However, IFNλR1-deficient (*Ifnλr1*^-/-^) mice were not preferentially susceptible to mCMV infection *in vivo* during acute infection after systemic or mucosal challenge, or during virus persistence in the mucosa. Instead, our studies revealed that IFNλ influences NK cell responses during mCMV infection. *Ifnλr1*^-/-^ mice exhibited defective development of conventional interferon-gamma (IFNγ)-expressing NK cells in the spleen during mCMV infection whereas accumulation of granzyme B-expressing NK cells was unaltered. *In vitro*, development of splenic IFNγ^+^ NK cells following stimulation with IL-12 or, to a lesser extent, IL-18 was abrogated by IFNλR1-deficiency. Thus, IFNλ regulates NK cell responses during mCMV infection and restricts virus replication *in vitro* but is redundant in the control of acute and persistent mCMV replication within mucosal and non-mucosal tissues.

## Introduction

The β-herpesvirus human cytomegalovirus (HCMV) is typically controlled by immune-competent individuals. However, HCMV causes disease in immune-suppressed adults such as transplant recipients, and in immunologically immature children following congenital infection. HCMV infects multiple tissues within the host and is transmitted horizontally via urine, breast milk and mucosal secretions including saliva [1]. Thus, understanding how CMV is controlled in peripheral tissues and identifying the factors that regulate these responses may inform therapeutic strategies.

Cytokines are important regulators of antiviral immunity during CMV infection (reviewed in [2]). The interleukin-10 (IL-10) cytokine superfamily consists of nine members that exhibit diverse functions ranging from immune regulation to tissue protection [3]. The importance of this superfamily in CMV pathogenesis is indicated by the evolutionary acquisition by HCMV [4] (and other herpesviruses [5, 6]) of IL-10 orthologues that suppress immune activation. Moreover, the rhesus CMV IL-10 orthologue restricts inflammation at the site of infection whilst suppressing long-lived virus-specific immunity *in vivo* [7]. Studies in the murine CMV (mCMV) model of infection, which recapitulates many aspects of HCMV tropism, immunity and pathogenesis [8] although notably not the acquisition of an obvious IL-10 orthologue, has highlighted the importance of cellular IL-10 in modulation of antiviral immunity during CMV infection *in vivo*. mCMV induces IL-10 [9], and the IL-10-IL-10R pathway inhibits virus-specific immunity and control of mCMV persistence in mucosal tissue [10–14] whilst limiting pathology and activation-induced NK cell death during acute infection [10, 15–17]. Additionally, the IL-10-related cytokine IL-22 is induced upon mCMV infection [18, 19] and restricts mCMV replication during acute infections of peripheral tissues via the recruitment of antiviral neutrophils [18]. Thus, IL-10 family members significantly influence CMV pathogenesis *in vivo*.

Interferon lambdas (IFNλ1–4, type III IFNs) are IL-10 superfamily members that exhibit functional parallels to type I IFNs with respect to induction of antiviral cellular immunity. In experimental models, IFNλ limits replication of numerous viruses (reviewed in [20]), including herpesviruses [21, 22]. IFNλ receptor (IFNλR) primarily signals in epithelial cells [23] and subsequently affords robust protection from viruses that target mucosal surfaces [24–28]. Given the importance of mucosal CMV infection in dissemination and pathogenesis, we investigated the mCMV model of infection to ascertain whether IFNλ influences the outcome of acute and persistent cytomegalovirus infection in mucosal and other peripheral tissues *in vivo*.

## Materials and methods

### Mice, infections and ethics

Smith strain murine cytomegalovirus (mCMV) originally obtained from the ATCC was generated following *in vivo* propagation in weanling BALB/c mice and purification of salivary gland-derived virus performed by spinning organ homogenate over a sorbital gradient, as previously described [18]. *Ifnlr1*^*tm1a(EUCOMM)Wtsi*^ (*Ifnλr1*^-/-^) mice were generated using gene targeting as part of the International Knockout Mouse Consortium (www.knockoutmouse.org) using high throughput methods as described by Skarnes *et al*., 2011 [29]. *Ifnλr1*^-/-^ and age/sex-matched wild type C57BL6/n mice were bred in-house at the Wellcome Trust Sanger Institute (WTSI) research support facility. Mice were infected with either 3 x 10^4^ or 5 x 10^4^ PFU mCMV (i.p.). In some experiments mice were infected with 1 x 10^4^ mCMV (i.n) under isofluothane anesthesia. Virus load in homogenized tissues was measured by plaque assay using 3T3 cells [18]. *In vitro* infections were performed in 3T3 and BNLCL2 cell lines (ATCC). Cells were treated with IFNλ2 (IL-28A) (Peprotech), IFNα or IFNβ (PBL Assay Science) for 24 hours prior to infection with Smith strain mCMV. After 4 days of infection, virus was assessed by plaque assay [18].

### Ethics statement

All mice experiments were performed under the UK Home Office-approved project Licence (Reference: PPL 80/2596) at the Wellcome Trust Sanger Institute research support facility. Isofluothane was used for anesthesia (for intranasal infections) and all mice were sacrificed according to UK Home Office guidelines.

### Assessment of *in vivo* immune responses

Splenocytes were isolated as previously described [16]. Liver leukocytes were isolated by passing leukocytes through a 70μM sieve prior to cell purification over a percoll gradient. NK cell responses were measured as previously described [16], with additional direct *ex vivo* assessment of intracellular granzyme B NK cell expression (Biolegend). Assessment of neutrophils and virus-specific T cell responses (quantified by detecting peptide-specific cytokine production) have been previously described [12, 14, 18]. IL-12 p70 (Biolegend), IL-18 (Thermo Fisher), IFNγ (Biolegend) and IFNλ2/3 (IL-28A/B, R&D Systems) protein was measured using ELISAs.

### Measured for *Ifnλr1* gene expression

RNA was extracted from cells using RNAeasy mini kit (Qiagen). Genomic DNA was eliminated from the samples using the Turbo DNA-free^™^ kit (Ambion) prior to cDNA synthesis (Applied Biosystems). Gene expression was measured by quantitative reverse transcription PCR using a QuantStudio^™^ 3 thermal cycler (Thermo Fisher Scientific) and iTaq Universal SYBR^®^ Green supermix (Bio-rad laboratories) using primers for Ifnλr1 (Forward: 5’-GTG ACC TAT TTC GTG ACC TAC C—3’, Reverse: R 5’-CTG CCT GTA CTC GTC CTT TG—3’) and β-actin (Forward: 5’-TGC AGA TTC CTC TCC AGC AA—3’, Reverse: 5’-GTC TTC ACC CCC TGA AAC CA—3’).

### *In vitro* NK cell assays

For analysis of NK cell function, splenocytes were isolated from naïve *Ifnλr1*^-/-^ mice and corresponding WT controls, plated in R10 medium supplemented with IL-12, IL-18 or IL-12/18 +/- IL-28a (10ng/ml, all Peprotech; 1 x 10^6^ cells per condition), and incubated for 5h with the addition of monensin (BD Biosciences). Cells were then incubated with Fc block (BioLegend), surface stained with anti-NK1.1 and anti-CD3ε, and then washed with DPBS (Gibco) and stained with zombie aqua dye (BioLegend). Surface-stained cells were then fixed and permeabilized using BD Cytofix/Cytoperm, and stained with anti-IFN-γ (BioLegend). To detection IFNλ-induced pSTAT1 activation in NK cells, splenocytes from naïve and mCMV infected mice were incubated for 1–6 hours with or without 10000U/mL IFN-β or 50ng/mL IFN-λ2. Cells were then surface-stained and phosphorylated STAT1 (pSTAT1) was detected according to the manufacturer’s instructions (BD Biosciences). Cells were analyzed on a Becton Dickinson FACsAriaIII using FACS Diva software (v8) or using an Attune NxT Flow Cytometer. Data were subsequently analysed using FlowJo software version 10.2.

### Statistics

Statistical significance was assessed using Mann-Whitney U for paired analysis of viral-load data whereas students T-test was used to analyze paired flow cytometry and ELISA data. 1-Way ANOVA was used to determine statistical significance where more than 2 groups were assessed concurrently (*in vitro* virus replication). For all tests performed, p values are reported as *≤0.05, **≤0.01, and ***≤0.001.

## Results and discussion

### IFNλ restricts mCMV replication *in vitro*

**Fig 1:**
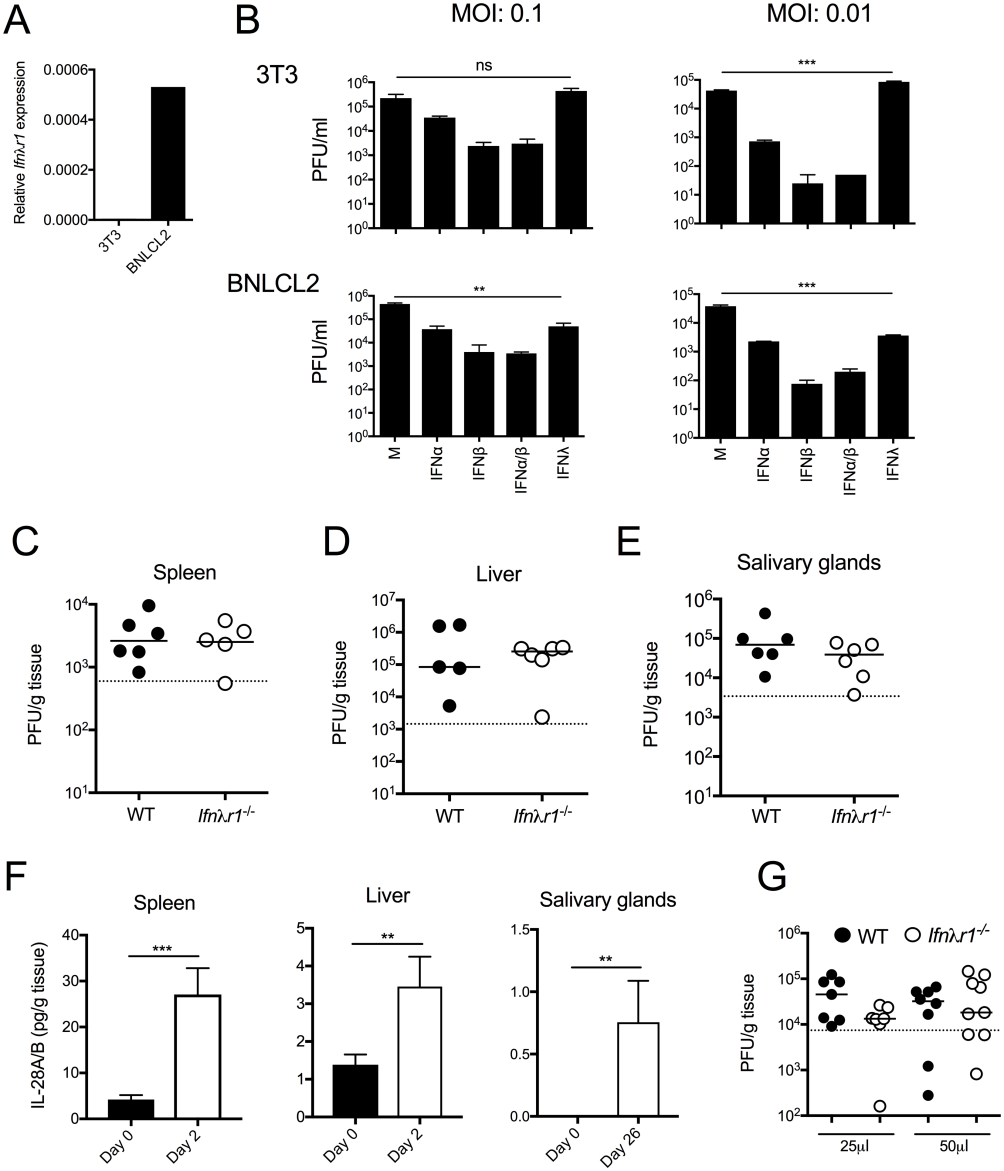
IFNλ can restrict mCMV replication *in vitro*. (A) *Ifnλr1* expression by 3T3 and BNLCL2 cells was determined by qPCR. (B) 3T3 (top) and BNLCL2 (bottom) cells were incubated with/without 50U/ml IFNα and/or IFNβ, or 50ng/ml IFNλ2 (IL-28A) for 24hrs and infected with mCMV at multiplicities of infection (MOI), as stated in the figure. After 4 days, infectious virions in supernatant were quantified by plaque assay. Statistical significance of PFU in IFNλ2-treated versus control cells is shown. Virus load in spleen (C), liver (D) and salivary glands (E) of WT and *Ifnλr1*^*-/-*^ mice was assessed 4 (D&E) and 33 (E) days p.i. (F) IFNλ2/3 protein in spleen (left), liver (middle) and salivary glands (right) was measured at day 0 and 2 days p.i (spleen and liver) or 0 and 26 days p.i (salivary glands). Results are shown as mean + SEM of 3–7 mice/group. (G) WT and *Ifnλr1*^*-/-*^ mice were infected (i.n) with mCMV in a volume of 25μl or 50μl and after 4 days, lung infectious viral load was quantified by plaque assay. Statistical significance was assessed using 1-way ANOVA (B) or Mann Whitney-U (C-E, G) or students T-Test (F) and is depicted where appropriate. Panel G represents merged data from two experiments whereas all other data represent at least two biological replicates performed separately.

We first assessed whether IFNλ directly influences mCMV replication, using murine cell lines representative of embryonic liver cells (BNLCL2) or fibroblasts (3T3) that expressed or did not express IFNλR, respectively (Fig 1A). Cells were pre-treated for 24 hours with or without IFNλ2, or as a positive control for cytokine-mediated control of mCMV replication, IFNα and/or IFNβ. In accordance with receptor expression data (Fig 1A), IFNλ did not impact upon mCMV replication in 3T3 cells following infection with a range of infection inoculums (Fig 1B). However, incubation of BNLCL2 cells with IFNλ prior to mCMV infection led to a reduction of replicative-competent virions in supernatant by ~1 log (Fig 1B). Induction of IFNλR in human astrocytes enables IFNλ-mediated control of HCMV replication *in vitro* [30]. Thus, our data is consistent with the conclusion that IFNλ is capable of restricting human and mouse cytomegalovirus infection within IFNλR-expressing cells.

### IFNλ does not impinge upon mCMV replication *in vivo*

We next studied IFN lambda receptor deficient mice (*Ifnλr1*^-/-^) to determine whether IFNλ modulated mCMV replication *in vivo*. Upon systemic (i.p.) challenge, we observed no effect of IFNλR1 deficiency on control of acute mCMV replication in the spleen or liver (Fig 1C and 1D), in accordance with previously published observations [31]. Although IFNλ restricted mCMV replication in a murine liver-derived cell line (Fig 1B), murine hepatocytes respond poorly to IFNλ [32]. However, epithelial cells are responsive to IFNλ *in vivo* [23]. Thus, we examined the impact of IFNλR1 deficiency on mCMV persistence in glandular epithelial cells within salivary glands. Again, *Ifnλr1*^-/-^ mice exhibited no defect in control of mCMV (Fig 1E). The absence of an antiviral function of IFNλ was not attributed to the lack of cytokine induction *in vivo* as significant expression was observed during acute and chronic infection (Fig 1F). Thus, we concluded that IFNλ does not contribute to the control of mCMV *in vivo* following systemic infection.

IFNλ restricts viral replication within the mucosa (reviewed in [20]). Using an established intranasal mucosal challenge model of mCMV infection [33–35], we investigated whether IFNλ restricted primary mucosal mCMV infection. We challenged adult mice with mCMV either in 25μl or 50μl volumes, reasoning that 25μl would restrict mCMV delivery to the nose and not trachea and thus serve to specifically probe the impact of IFNλR signaling on control of initial mCMV replication within the nasal cavity. As shown in Fig 1G, we observed no impact of IFNλR1 deficiency on mCMV infection of lung tissue following mCMV infection using either volume (Fig 1G). Thus, although the caveat exists that the unresponsive nature of murine hepatocytes to IFNλ may mask a possible antiviral role for IFNλ in controlling CMV replication within the liver, overall our data supports the conclusion that IFNλR signaling plays no significant role in controlling mCMV replication *in vivo*.

### IFNλ does not influence the development of mCMV-specific T cell immunity

**Fig 2:**
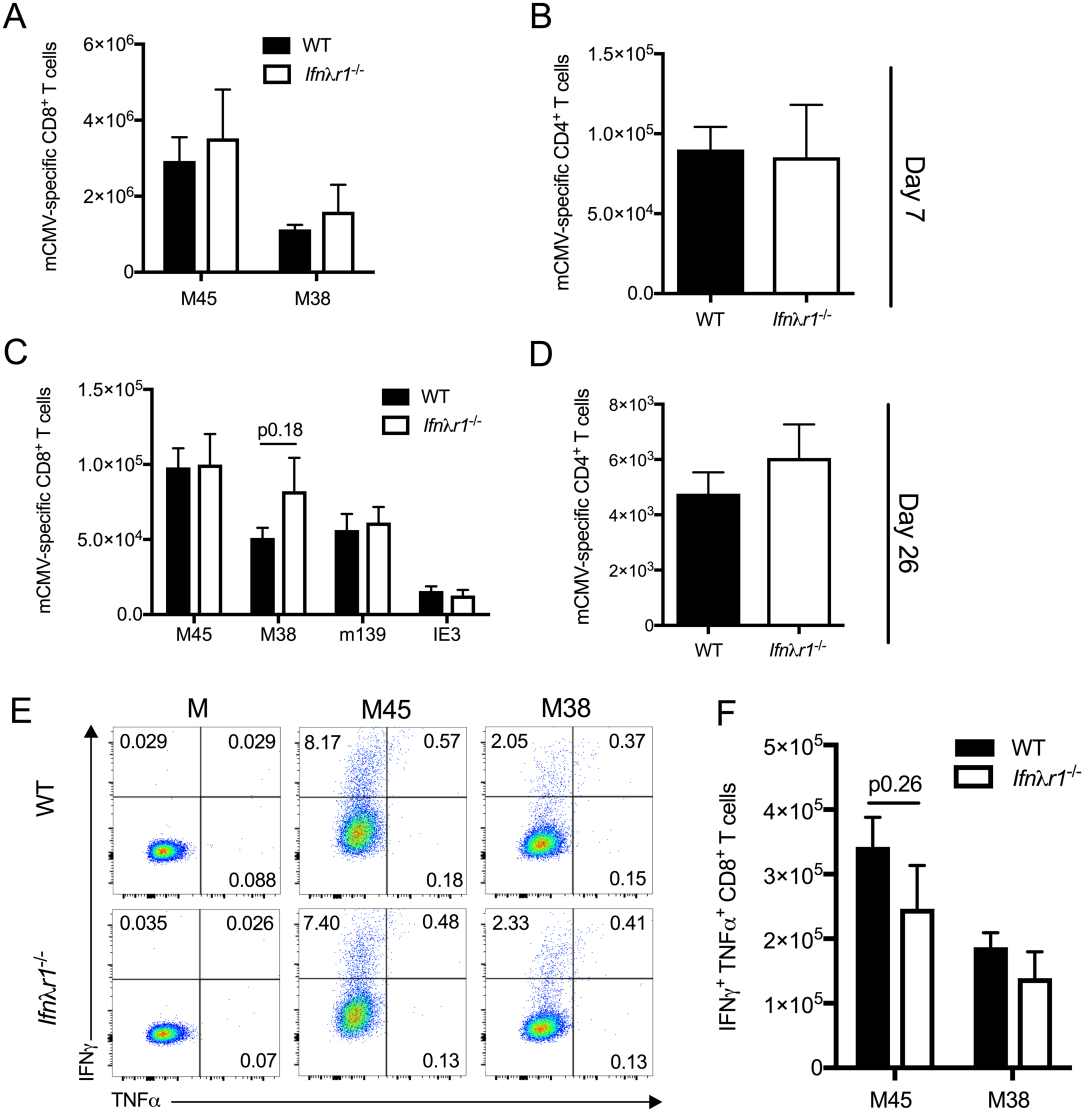
IFNλR signaling does not influence mCMV-specific T cell responses *in vivo*. mCMV-derived peptide-specific CD8^+^ (A&C) and CD4^+^ (B&D) T cell responses in WT and *Ifnλr1*^*-/-*^ mice infected for 7 (A&B) or 26 (C&D) days were quantified *ex vivo* following peptide re-stimulation. Mean + SEM of 6–7 mice/group is shown and represent 2–3 experiments. Statistical significance was tested using an unpaired student’s T-test. (E) Representative bivariate FACS plots of IFNγ and/or TNFα expression by CD8^+^ T cells stimulation with media alone (M) or 2μg/ml MHC class I restricted peptide derived from mCMV M45 (HGIRNASFI) or M38 (SSPPMFRVP) proteins. % positive cells are shown in the plots. Data are representative of 6 mice/group. (F) Mean + SEM from 6 mice/group is shown. Results represent 2 separate experiments.

IFNλ has been implicated in shaping adaptive immunity [20]. Studies of lymphochoriomeningitis infection revealed that IFNλ can restrict effector T cell responses and memory development following acute virus infection whilst paradoxically promoting T cell responses during virus chronicity [36]. Using intracellular staining for IFNγ following ex vivo stimulation with mCMV-derived peptides, we measured mCMV-specific CD8^+^ and CD4^+^ T cell responses during acute (d7 p.i) and persistent (d26 p.i) infection. We detected no discernable differences in the accumulation of functional virus-specific CD4^+^ or CD8^+^ T cells between WT and *Ifnλlr1*^*-/-*^ mice at either time-point (Fig 2A–2D). Furthermore, IFNλR1 deficiency did not influence the frequency of mCMV-specific T cells that co-express TNFα following re-stimulation *ex vivo* (Fig 2E and 2F). Therefore, although these data do not preclude a role for IFNλ in modulating T cell and humoral immunity during the chronic/latent phase of mCMV infection, these results demonstrate that IFNλ does not alter the magnitude or quality of mCMV-specific T cell responses during acute and persistent mCMV infection.

### IFNλR1 promotes splenic IFNγ^+^ NK cell responses during mCMV infection

**Fig 3:**
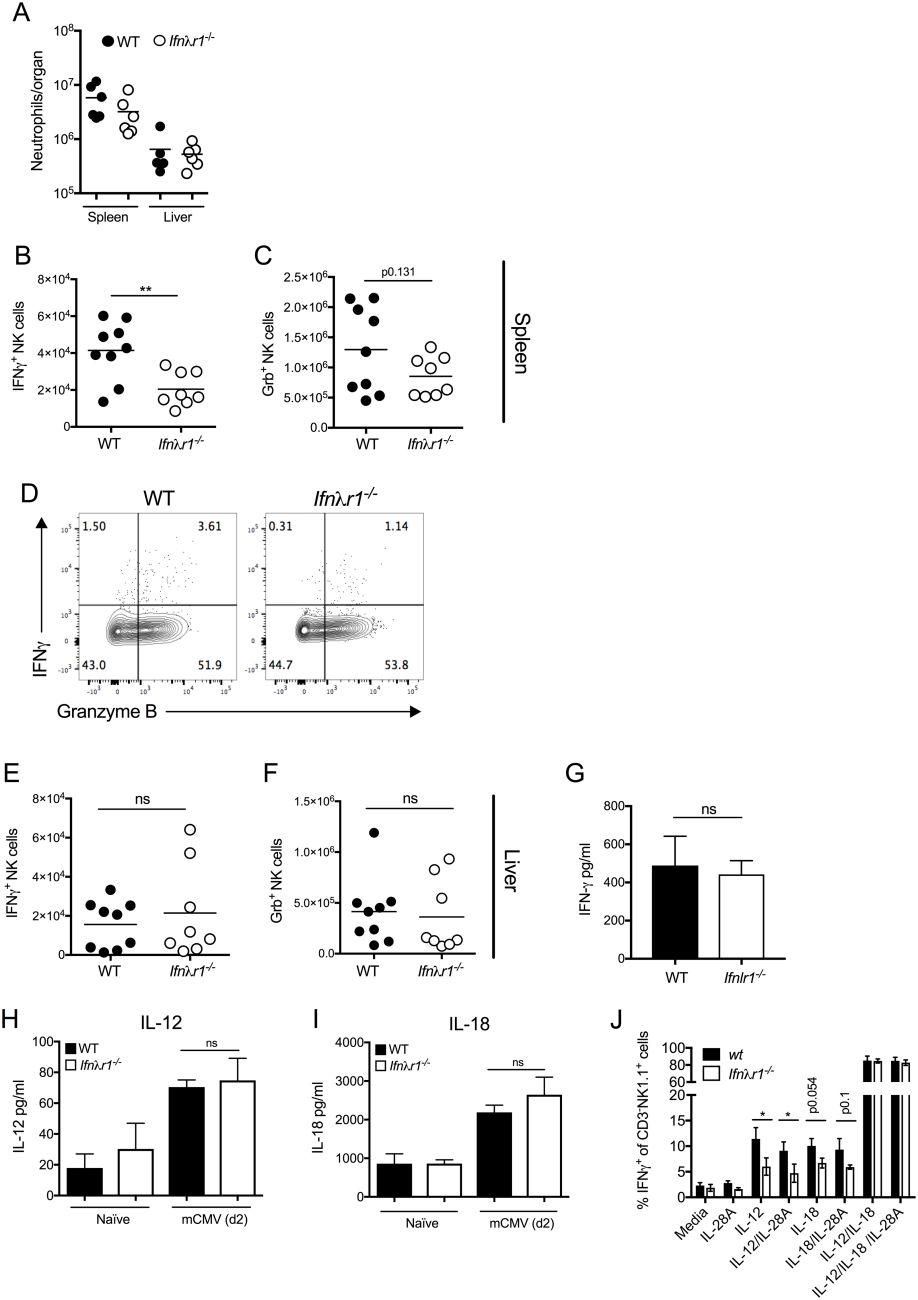
IFNλ promotes IFNγ^+^ NK cell responses. (A) Neutrophil numbers in the spleens and livers of mCMV-infected WT and *Ifnλr1*^*-/-*^ mice was assessed 4 days p.i. IFNγ^+^ (B&D) and granzyme B^+^ (C&E) NK cell accumulations in spleens (B-C) and livers (E&F) were quantified day 4 pi. Individual mice + mean is shown from 2 independent experiments and represent 4 (liver) or 5 (spleen) experiments in all. (D) Representative bivariant FACS plots of IFNγ versus granzyme B expression by live NK1.1^+^CD3^-^ cells 4 days post-infection. Plots show concatenated samples from 4 (WT) and 6 (*Ifnλr1*^*-/-*^) mice. Results represent 3 experiments. (G) IFNγ protein in the serum was measured 4 days pi. Mean + SEM of 3–4 mice/group is shown. (H&I) IL-12 (H) and IL-18 (I) protein was measured in naïve spleen tissue or after 2 days of mCMV infection. Mean + SEM of 3 (naïve) or 7 (infected) mice is shown. (J) IFNγ expression by NK cells stimulated with IL-12, IL-18 or IL-12/IL-18 +/- IL-28A was detected by flow cytometry. Data represent the mean +/- SD for 3 mice per group. All experiments were performed at least twice.

We next investigated whether IFNλ modulates innate immune activation during mCMV infection. Neutrophils limit mCMV replication and pathogenesis [18, 37], and IFNλ has been reported to directly modulate neutrophil responses *in vivo* [38, 39]. However, neutrophil accumulation in the spleen and liver during mCMV infection was unaltered in *Ifnλr1*^*-/-*^ mice (Fig 3A). In contrast, however, studies of NK cell responses revealed a marked reduction in NK cells from spleens from *Ifnλr1*^*-/-*^ mice during acute mCMV infection that spontaneously produce IFNγ ex-vivo (Fig 3B). Defective IFNγ^+^ NK cell accumulation was not defined by a broad deficit in NK cell responses in *Ifnλr1*^*-/-*^ mice as, although we observed a trend in reduced accumulation of cytotoxic (granzyme B^+^) splenic NK cells in *Ifnλr1*^*-/-*^ mice, this was not statistically significant (Fig 3C and 3D). NK cell populations that are present in the liver form a distinct lineage from splenic NK cells [40]. Interestingly, we observed no statistically significant impact of IFNλR deficiency on the accumulation of IFNγ^+^ or granzyme B^+^ hepatic NK cells (Fig 3E and 3F) nor did we detect a defect in systemic IFNγ protein concentrations (Fig 3G), suggesting that IFNλ preferentially promoted the development of IFNγ^+^ conventional splenic NK cell responses during mCMV infection rather than broadly impacting upon NK cell responses in other tissues and influencing systemic cytokine production.

We next examined the mechanism(s) through which IFNλ regulates IFNγ^+^ NK cells. IL-18 and IL-12 promote these responses during mCMV infection [41–43]. However, IL-12 and IL-18 protein concentrations were comparable in spleen supernatants from WT and *Ifnλr1*^*-/-*^ mice post-infection (Fig 3H and 3I), suggesting that IFNλ did not promote IFNγ^+^ NK cell responses indirectly via the regulation of IFNγ-inducing cytokines. Instead, incubating splenocytes from naïve mice with IL-12 revealed impaired development of *Ifnλr1*^*-/-*^ IFNγ^+^ NK cells, as assessed by flow cytometry after 5hrs (Fig 3J). A similar, albeit not significantly significant, trend was observed in splenocytes incubated with IL-18 (Fig 3J). These data support the conclusion that IFNλR signaling promotes NK cell responsiveness to IFNγ-inducing cytokines.

Souza-Fonseca-Guimaraes et al [31] previously reported no impact of IFNλR deficiency on cytokine-induced IFNγ secretion by purified NK cells. In our experiments, we studied whole splenocytes and examined IFNγ^+^ NK cell generation by flow cytometry and not ELISA, and at earlier time-points than Souza-Fonseca-Guimaraes and colleagues. Thus, variations in experimental designs may explain these disparate findings. Importantly, however, we saw no influence of IFNλR signaling on IFNγ^+^ NK cell development that was induced following co-incubation of splenocytes with IL-12 and IL-18 (Fig 3H). Given that Souza-Fonseca-Guimaraes and colleagues performed assays using purified NK cells that involved co-incubation of multiple cytokines, our data suggest that the influence of IFNλR1 on IFNγ^+^ NK cell development may be redundant in situations where cells receive strong stimuli via concurrent stimulation of multiple cytokine receptors. Indeed, the observation that IL-12 and IL-18 are often co-expressed during mCMV infection [41] may explain why hepatic NK cell responses and systemic IFNγ responses were unaltered in *Ifnλr1*^*-/-*^ mice. Whether early IL-18 production in the spleen in the absence of robust IL-12 secretion following systemic mCMV infection [41] is responsible for the observation that *Ifnλr1*^*-/-*^ mice mount reduced IFNγ^+^ NK cell responses in this tissue, is unclear.

IFNλR1 has been shown to directly induce IFNγ production by NK cells *in vivo* [31], although whether direct IFNλR signaling occurs in human NK cells and induces IFNγ expression remains controversial [44, 45]. In agreement with Souza-Fonseca-Guimaraes et al, we found that IFNλ (IL-28A) protein appeared not to directly increase IFNγ^+^ NK cell development in our studies (Fig 3H), suggesting that IFNλ does not act as a co-factor for IL-12 and/or IL-18 induction of IFNγ^+^ NK cells, at least *in vitro* in the time-frame examined. Furthermore, IFNλ failed to induce STAT1 phosphorylation in NK cells derived from naïve or mCMV-infected mice (S1 Fig). Instead, the observation that *Ifnλr1*^*-/-*^ NK cells from naïve mice are less responsive to IL-12 and IL-18 implies that although NK cell repertoires are comparable in WT and *Ifnλr1*^*-/-*^ mice [31], IFNλ may promote NK cell responsiveness to IL-12/IL-18 prior to stimulation with these cytokines. It is feasible that this occurs during NK cell development or in either a different anatomical location or over a different timescale to that studies herein. Alternatively, our data does not preclude the possibility that dysregulated IL-10R2 expression, signaling and/or distribution that may occur in *Ifnλr1*^*-/-*^ mice as a consequence of genetic deletion of this partner of IL-10R2 may impact on NK cell responses.

Overall our data suggest that although IFNλ can directly restrict mCMV replication *in vitro*, this is non-essential during the acute and mucosal persistence phases of *in vivo* infection. IFNλ predominantly protects mucosal tissue from viral infections [24–28]. Upon mucosal challenge with mCMV, rapid viral dissemination into the spiral ganglia can occur that is independent of local mCMV replication [35]. Thus, initial control of virus replication within mucosal tissue may be unimportant for host protection. Alternatively, given similarities in signaling pathways induced by IFNλR and type I IFN receptor [46], type I IFN may render IFNλR signaling redundant in immune control of mCMV replication *in vivo*. Furthermore, mCMV may blunt direct antiviral activities of IFNλ. Indeed, IFNλ induces STAT2 activation [46] and the mCMV-encoded pM27 promotes proteasomal degradation of STAT2 [47]. Further, our data does not preclude the possibility that mCMV may interfere with IFNλR abundance and/or localization. Therefore, although IFNλ is capable of limiting mCMV replication in *in vitro assays*, it is possible that viral immune evasion mechanisms may blunt this antiviral cytokine pathway *in vivo*.

Rather than acting as an antiviral cytokine during mCMV infection *in vivo*, our data revealed that IFNλ exhibits immune modulatory activity by regulating the accumulation of functional NK cells. IFNλ did not impact broadly on NK cell responses, in contrast to the induction of NK cell proliferation by IL-28B during influenza infection [48]. Instead, IFNλ preferentially promoted the development of IFNγ^+^ conventional NK cells in the spleen, and our data implied that this occurred via regulation of NK cell responsiveness to IL-12 and, possibly, IL-18. IFNλ specifically induced NK cell responses that express IFNγ but not granzyme B and did not impact on systemic IFNγ secretion. This selective influence on NK cell responses may explain why IFNλR deficiency had no impact on control of virus replication. Overall, our data demonstrate that IFNλ modulates conventional NK cell responses in response to systemic virus infection.

## Supporting information

S1 Fig IFNλ does not induce STAT1 phosphorylation in NK cells from naïve or mCMV-infected mice. Splenocytes from naïve mice (A&B) or from mice infected (i.p) for 4 days with mCMV (C&D) were stimulated with/without IFNβ or IFNλ and after 1, 3 and 6 hours, STAT1 phosphorylation was measured. (A&C) STAT1 phosphorylation by NK1.1^+^CD3^-^ is expressed as Median fluorescent intensity (MFI) and mean + SEM of 5 mice is shown. (B&D) Representative histogram overlays of STAT1 phosphorylation in viable NK1.1^+^CD3^-^ cells after 1 hour of stimulation with/without cytokines. Data are representative of 5 separate mice from infected or naïve groups.
(TIFF)
