# Supplementary figures and images for: Interferon lambda is required for interferon gamma-expressing NK cell responses but does not afford antiviral protection during acute and persistent murine cytomegalovirus infection
Source: PLoS One. 2018 May 16;13(5):e0197596. doi: 10.1371/journal.pone.0197596 (PMC5955543; doi:10.1371/journal.pone.0197596)

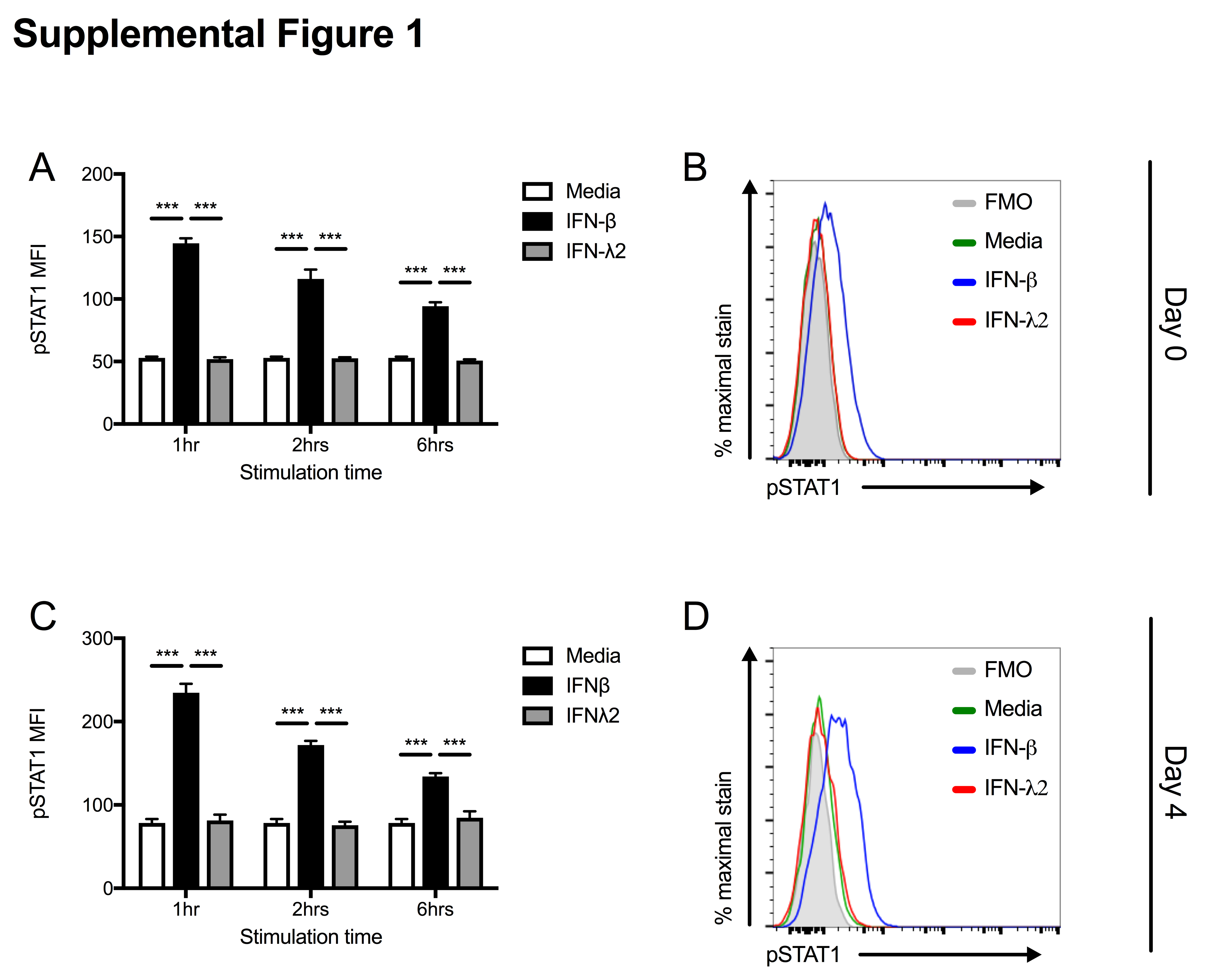

Supplement: S1 Fig — Splenocytes from naïve mice (A&B) or from mice infected (i.p) for 4 days with mCMV (C&D) were stimulated with/without IFNβ or IFNλ and after 1, 3 and 6 hours, STAT1 phosphorylation was measured. (A&C) STAT1 phosphorylation by NK1.1+CD3- is expressed as Median fluorescent intensity (MFI) and mean + SEM of 5 mice is shown. (B&D) Representative histogram overlays of STAT1 phosphorylation in viable NK1.1+CD3- cells after 1 hour of stimulation with/without cytokines. Data are representative of 5 separate mice from infected or naïve groups. (TIFF) [file pone.0197596.s001.tiff]
